# High taurine supplementation in plant protein-based diets improves growth and organoleptic characteristics of European seabass (*Dicentrarchus labrax*)

**DOI:** 10.1038/s41598-020-69014-x

**Published:** 2020-07-23

**Authors:** Yannis Kotzamanis, Theofania Tsironi, Andreas Brezas, Kriton Grigorakis, Vassiliki Ilia, Ioannis Vatsos, Nicholas Romano, Jan van Eys, Vikas Kumar

**Affiliations:** 1 Fish Nutrition Lab, Institute of Marine Biology, Biotechnology and Aquaculture, Hellenic Centre for Marine Research (HCMR), Agios Kosmas, Hellinikon, 16777 Athens, Greece; 2 Food Process Engineering Laboratory, Department of Food Science and Human Nutrition, Agricultural University of Athens, 11855 Athens, Greece; 3 Department of Animal and Veterinary Science, Aquaculture Research Institute, University of Idaho, Moscow, ID 83844 USA; 4 Faculty of Biosciences and Aquaculture, Nord University, Post Box 1490, 8049 Bodø, Norway; 5 Department of Aquaculture and Fisheries, University of Arkansas at Pine Bluff, Pine Bluff, USA; 6 GANS Inc., 24 Av. de La Guillemotte, 78112 Fourqueux, France

**Keywords:** Physiology, Feeding behaviour

## Abstract

Plant-based proteins are increasingly being used in aquafeeds, but one of the limitations is taurine deficiency that can be especially detrimental for carnivorous fish. In this study, taurine supplementation in high plant protein diets (low fish meal, 15%) was investigated on the growth performance and fillet organoleptic characteristics of European seabass (*Dicentrarchus labrax*), juveniles (78 ± 0.4 g). Five diets were formulated to contain 0 (C−), 5 (T5), 10 (T10) or 20 (T20) added taurine (g/kg), while a control diet (C+) included two-fold higher amount of fishmeal (30%) with no taurine supplementation. Seabass fed the T20 or C+ diet showed similar growth, which was significantly higher compared to those in the C-treatment. Histological examination of the muscle, liver and intestine were similar among treatments. No effect on spoilage bacterial growth or production of total volatile basic nitrogen was observed. Taurine supplementation significantly reduced fillet drip loss, while the fillets of European seabass fed the T10 and T20 diets showed significant higher and lower hardness and adhesiveness values, respectively. Moreover, fillet chewiness was also found to be significantly higher in fish fed the T20 diet compared to C+ and C− diets. Overall, it seems that high dietary taurine supplementation acted as a growth promoter and concurrently improved significantly the postharvest quality characteristics of seabass, which may be attributed to its role in maintaining cell membrane integrity and permeability.

## Introduction

Fish meal (FM) for many years was the major protein source for use in aquafeeds, however, due to its dependence on finite wild-fish stocks^1^ this resource is increasingly being replaced with various plant-based sources in aqua-feeds, an approach which supports aquaculture sustainability. The consequences of the high FM replacement have been reported to induce deleterious effects to the fish performance, especially in carnivorous species^2^. This is often attributed to many different causes, such as the presence of anti-nutritional factors present in plant-origin ingredients^3^, their imbalanced amino acid profile^4^ as well as to the absence of other nutrients, such as taurine^5^.

Taurine is a beta-amino acid, but unlike others, it cannot be used for protein synthesis or energy^6^. However, taurine is involved in many important physiological processes that include membrane stability, osmoregulation, and immunity^2^. Moreover, taurine is preferentially conjugated with bile acids to form bile salts (i.e. taurocholic acid and taurochenodeoxycholic acid) that facilitate lipid digestion^7^. Fish have the ability to synthesize taurine from methionine and cysteine via cysteinesulfinic acid decarboxylase, but is generally inferior compared to mammals^8^. Considering that plant proteins contain little to no taurine, supplementation of this nutrient to the fish diets may be of high importance ^5,9–13^.

European seabass (*Dicentrarchus labrax*, *L*.) is one of the two the main cultured marine finfish species in the European aquaculture and is considered as a high value species farmed in the Mediterranean region. Research on the effects of taurine supplementation in the plant protein-based diets of European seabass (*Dicentrarchus labrax*) has been controversial. Coutinho et al.^14^ found no beneficial effect on growth or feed efficiency when taurine was supplemented at 10 g/kg in the diet of European seabass juveniles (~ 7 g initial mean body weight). In an earlier study, relatively low taurine levels of 2 or 3 g/kg significantly improved the growth of European seabass fry (0.8 initial mean body weight) compared to those fed diets with 0 or 1 g/kg taurine supplementation^15^. Similarly, in a low FM diet, dietary taurine supplementation of 0.5% significantly improved European seabas*s* juvenile growth (55 g initial mean body weight)^16^. Recently, Kotzamanis et al.^17^ found that taurine supplementation (1%) in a high-soy diet with moderate FM inclusion (25%) impacted significantly the organoleptic properties of seabass fillet but without substantially affecting their growth performance.

A research area of high interest is considered to be the potential effects of taurine supplementation in plant protein-based diets on the post-harvest quality of fish fillet^17,18^. In most cases, plant-based diets have little to no effect on the texture or various organoleptic qualities in fish ^19–21^. However, de Francesco et al.^22^ did find that a plant protein mixture negatively affected the texture and organoleptic qualities of rainbow trout (*Oncorhynchus mykiss*) compared to those fed a FM-based diet. However, the effects that taurine supplementation in high plant—low FM diets might have on post-harvest fillet quality of European seabas*s* have not been thoroughly investigated. The aim of the present study was to investigate the effects of incremental taurine supplementation in a high plant—low FM diet, on the growth performance, and post-harvest fillet organoleptic characteristics of European seabass.

## Materials and methods

### Ethical permits

All animal handling and sampling procedures were conducted in accordance with Greek (PD 56/2013) and EU (Directive 63/2010) laws and regulations regarding the protection of animals used for scientific purposes. Furthermore, the aqua-laboratories of the Hellenic Centre for Marine Research in Athens are certified by the Greek Veterinary authorities for the breeding and use of animals for scientific purposes (ΕL-25-BIO-037). The use of experimental fish (European seabass, *Dicentrarchus labrax*) was according to the scientific research protocols of Greek Veterinary authorities that were approved by Centre for Marine Research in Athens, Greece in accordance with all relevant local and/or international animal welfare laws, guidelines and policies.

### Experimental diets

Five isonitrogenous and isoenergetic diets were formulated. A positive control diet (C+) was formulated to contain 300 g/kg FM and a plant protein mixture without taurine supplementation. Three other experimental diets were formulated to contain a low FM inclusion (150 g/kg), and a high amount of plant protein mixture with three levels of taurine supplementation, 5 g/kg, 10 g/kg and 20 g/kg corresponding to T5, T10 and T20 diets, respectively. Finally, a negative control diet (C−) was formulated to have identical composition to the previous three diets, but without any taurine supplementation. The experimental feeds were manufactured at BIOMAR’s Tech Centre in Brande, Denmark. Diets (3.5 mm pellets) were prepared by cooking-extrusion. Following production, pellets were bagged and shipped to the HCMR’s facilities in Athens. The ingredients and chemical composition of the diets are provided in Table 1.

**Table 1:** Dietary formulation and proximate composition (g per kg) of the experimental diets.

Ingredients	C+	C−	T 5	T 10	T 20
Fish meal^a^	300	150	150	150	150
Soya cake^b^	200	200	200	200	200
Soya protein concentrate^c^	0	120	120	120	120
Corn gluten	175.4	232.3	222.9	214.5	195.7
Wheat	157	100	102	104	109
Fish oil^a^	61	74	74	75	76
Rapeseed oil	79	75	76	76	77
Lysine^d^	0	4.28	4.38	4.49	4.7
Methionine^d^	0	1.14	1.28	1.42	1.71
Vitamin & mineral premix^d^	3	3	3	3	3
Monocalcium phosphate^e^	5.4	15.0	15.2	15.3	15.6
Taurine^f^	0	0	5	10	20
**Analyzed proximate composition**					
Dry matter	893	904	904	911	905
Crude protein	435	439	443	433	435
Crude lipid	179	171	174	176	171
Ash	56	56	55	55	56
Carbohydrate*	223	238	224	247	243
Gross energy (MJ kg^−1^)**	21.2	21.2	21.2	21.4	21.2
**Analysed amino acid composition (g/100 g feed)**					
Histidine	0.88	0.87	0.90	0.89	0.87
Taurine	0.20	0.11	0.58	1.06	2.02
Serine	2.15	2.18	2.18	2.23	2.14
Arginine	2.46	2.41	2.41	2.46	2.39
Glycine	1.95	1.73	1.72	1.76	1.70
Asx	4.44	4.50	4.48	4.55	4.46
Glx	8.48	8.90	8.82	9.00	8.62
Threonine	1.73	1.66	1.67	1.68	1.63
Alanine	3.02	2.93	2.89	2.95	2.79
Proline	2.56	2.69	2.66	2.72	2.56
Cystine	0.25	0.26	0.29	0.28	0.26
Lysine	2.77	2.77	2.79	2.86	2.82
Tyrosine	1.40	1.46	1.46	1.44	1.37
Methionine	0.84	0.75	0.79	0.75	0.74
Valine	2.08	2.00	1.97	2.01	1.93
Isoleucine	1.81	1.81	1.78	1.80	1.73
Leucine	4.31	4.48	4.43	4.51	4.25
Phenylalanine	2.02	2.12	2.11	2.14	2.04

^a^Supplied by Norsildmel Innovation AS.

^b^Purchased from Cargill.

^c^Supplied by Imcopa.

^d^DL-methionine, Lysine and L-Threonine and Vitamin/Mineral premix was supplied by Vilomix.

^e^Supplied by Yara International.

^f^Crystalline taurine was supplied by Omya Peralta GmbH.

*Calculated by difference: 100—(%protein + %fat + %ash + %moisture) (i.e. N-free extractives + crude fiber).

**Gross energy was calculated using combustion values for protein 23.6 MJ kg^−1^, lipid 39.5 MJ kg^−1^ and carbohydrate 17.2 MJ kg^−1^, respectively.

### Animals and experimental design

European seabass juveniles were obtained from a commercial fish farm owned by Selonda SA based near the town of Epidaurus, Peloponnesus, Greece. Fish were transferred to HCMR’s facility in Agios Kosmas, Athens, Greece. European seabass juveniles with an initial average body weight of 78 ± 0.4 g (mean ± SD) were assigned to 15 experimental cylindro-conical tanks (1,000 L), equipped with waste collectors, with 33 fish per tank, three tanks per diet. At the beginning of the experiment the initial population was individually weighed to the nearest 0.1 g. Before weighing, the fish were anaesthetized using MS-222 (50 mg/L).

Each tank was supplied with a through flow of natural seawater (salinity 35 ppt), at a relative renewal rate of 400 L h^−1^ and aerated to over 75% of oxygen saturation level. Water temperature during the experimental period was 27.6 °C ± 0.9, while the natural photoperiod cycle was followed. After acclimatization of fish to experimental tanks for 2 weeks, five fish from the initial population were randomly sampled and sacrificed using an overdose of anesthetic (MS-222). Then these fish were pooled, minced, freeze-dried and grounded for the analysis of initial whole-body composition. Each experimental diet was randomly allocated in triplicate groups. The fish were hand-fed to visual apparent satiation two times daily (09:00 and 15:00 h) six days a week. Uneaten feed was collected, dried and weighted after each meal and the daily consumption was recorded. The feeding trial was continued over a period of 90 days.

At the end of the feeding trial, all fish were weighed individually, while 10 fish were randomly sampled from each tank. Muscle samples from five fish were pooled and stored at once at 80 °C for measurements of proximate and amino acid composition, while livers and intestines from three of the above five sampled fish from each tank were used for histological examination. In addition, the perivisceral fat was also measured in the five sampled fish from each tank. The remaining five fish from each tank were pooled and analysed for whole body composition. Similarly, three more fish from each tank were sampled for texture analysis while an extra pool of nine fish per diet from different tanks was sampled from diets C+, C−, T1.0 and T2.0 for sensory analysis. Finally, 12 fish from diets C+, C−, T1.0 and T2.0 were also sampled for the shelf life study.

### Equations

The following parameters were evaluated:

- Survival (%): 100 − mortality.
- Total feed intake, (TFI) per fish = g DM feed/fish, where DM is the dry matter of the mean feed consumption per fish.
- Specific growth rate (SGR): 100 × {(ln FBW − ln IBW)/days}, where, IBW and FBW are the initial and final body weight, respectively.
- Relative feed intake, (FI) (%/d) = 100 × (TFI/ΙBW).
- Daily growth index, DGI (%) = (FBW^1/3^ − IBW^1/3^)/days × 100.
- Thermal growth coefficient, (TGC) = (FBW^1/3^ − IBW^1/3^) × (ΣD^0^)^−1^, where ΣD^0^ is the thermal sum (feeding days × average temperature, ºC).
- Feed conversion ratio (FCR) = dry feed consumed (g)/body weight gain (g).
- Protein efficiency ratio (PER) = body weight gain (g)/protein intake (g).
- Lipidosomatic Index (LSI, %) = 100 × (visceral fat (g)/body weight (g)).
- Hepatosomatic Index (HSI) = 100 × (liver weight (g)/body weight (g)).

### Proximate and amino acid composition

Samples of diets and lyophilized fish whole body as well as fish muscles from each tank were analysed for crude protein, total fat, ash and energy according to AOAC^23^. Moisture content was measured after drying the samples at 105 °C for 24 h, ash was determined after ignition at 500 °C for 12 h, crude protein (N × 6.25) by Kjeldahl method and total fat was estimated gravimetrically by Soxhlet extraction (for the determination of fish muscle lipid content the Folch’s procedure was used).

The amino acid composition of the diets (Table 1) and fillets was analyzed after acid hydrolysis (6 N, 110 °C, 24 h) and derivatization by AccQ-Tag™ Ultra according to the amino acid analysis application solution (Waters Corporation, Milford, MA, USA). DL-Norvaline (Sigma) 2.5 mM was used as an internal standard. UPLC was performed on an Acquity system (Waters Corporation) equipped with PDA detector and the detection wavelength was set at λ = 260 nm. The column used was a BEH C18 column (100 mm × 2.1 mm i.d., 1.7* μm*) from Waters. The flow rate was 0.7 ml/min and the column temperature were kept at 55 ºC. Peak identification and integration were performed by the software Empower v.2.0 (Waters) using an Amino Acid Standard H (Pierce) as an external standard. All analyses were performed in duplicate. In case that the values between replicates did not meet the standardized acceptance criteria based on the mean and standard deviation (< 5%), new duplicate analyses were performed according to established procedures. Tryptophan was not quantified due to its susceptibility to acid hydrolysis, while cysteine reacts with cysteine forming a disulfide bridge to produce cystine. Moreover, during acid hydrolysis procedure asparagine is converted to aspartate (ionic form of aspartic acid) and glutamine to glutamate (ionic form of glutamic acid), so the reported values for these amino acids (Asx and Glx) represent the sum of both amino acids.

### Texture analysis

Texture parameters were determined using a texture analyzer with a load cell of 5 kg (MODEL TA-XT2i, Stable Micro Systems, Godalming, Surrey, United Kingdom). A flat-ended cylinder of 20 mm diameter was selected to simulate the human finger. Constant penetration depth was applied on the fish flesh and penetration depth of 2.0 mm was selected as the maximum distance which could be applied without affecting the muscle structure by erupting and leaving a mark on the fish flesh. Double compression was applied to construct the texture profile analysis (TPA) parameters of the fillets of two different specimens. The cylinder approached the sample at the speed of 0.5 mm·s^-1^ and penetrated 2 mm into the fish flesh. Then the force was reduced and the sample was allowed to rebound for 5 s. The cylinder was pressed on the sample a second time, force-distance curves were recorded and analyzed using the Texture Expert Exceed Application (Version 2.64, Stable Micro Systems Ltd) and texture analysis parameters (hardness, springiness, cohesiveness, adhesiveness and chewiness) were calculated^25^.

### Sensory analysis

A descriptive analysis taste panel was conducted in Selonda S.A fish fillet plant, Epidaurus, Greece, in order to evaluate the colour, flavour, taste intensities and the textural characteristics (hardness, elasticity, cohesiveness, chewiness, and stickiness) of the studied fish groups. The textural characteristics that were evaluated were as those described by Szczesniak^26^ for solid foods. Also, the overall likeness of the fish fillets was evaluated. All of these organoleptic characteristics were evaluated on a 0–10 scale on steam-cooked (20 min steam cooking) whole fish fillets. The panel consisted of 20 persons selected among the Selonda S.A. personnel and with a short training in order to understand textural attributes.

### Shelf life analysis

Shelf life tests were conducted at two constant storage temperatures (0 and 5 °C), in order to test the recommended and non-recommended temperature conditions. Samples were stored aerobically (in unsealed pouches) with one fillet in each package. Shelf life evaluation was based on microbiological analysis (total viable count, *Pseudomonas* spp. and *Enterobacteriaceae* spp.), total volatile basic nitrogen (TVBN), drip loss and sensory evaluation of raw samples (appearance and odour). 10–12 samples in each series (analyzing alternatively single and double specimens to check repeatability) were analyzed.

#### Microbiological and total volatile basic nitrogen analyses

For microbiological enumeration, a 10 g sample was transferred to a sterile stomacher bag with 90 mL of sterilized Ringer solution (Merck, Darmstadt, Germany) and was homogenized for 60 s. Samples (0.1 mL) of tenfold serial dilutions of fish homogenates were spread onto the surface of the appropriate media in Petri dishes for enumeration of different spoilage bacteria^27^. Total aerobic count was enumerated on Plate Count Agar (PCA, Merck, Darmstadt, Germany) after incubation at 25 °C for 72 h. *Pseudomonas* spp. were enumerated on Cetrimide Agar (CFC, Merck, Darmstadt, Germany) after incubation at 25 °C for 48 h. For *Enterobacteriaceae* spp. enumeration the pour-plate method was used, using the Violet Red Bile Dextrose Agar (VRBD, Merck, Darmstadt, Germany), incubated at 25 °C for 48 h. Two replicates of at least three appropriate dilutions were enumerated. The microbial growth was modelled using the Baranyi Growth Model^28^. For curve fitting the in-house program DMfit (Inst. Food Research, Reading, U.K.) was used, and kinetic parameters such as the rate (k) of the microbial growth were estimated. Total volatile basic nitrogen analysis was conducted on a single TCA extraction by distillation in a Kjeldhal rapid distillation unit and titration with hydrochloric acid^29^.

#### Drip loss and sensory evaluation

For measurements of drip loss, pouches were reweighed with and without a fillet (two pouches for each diet and storage temperature). Before sampling, fish fillets were removed from the pouch leaving the drip and the pouch containing the drip was then weighed. Drip loss was computed from the weight of the drip (pouch containing the drip minus the pouch) and expressed as a percentage loss based on the initial sample weight. Readings from the two determinations for each condition were averaged^30^. A score of 5 for sensory scoring was judged as the lower limit of acceptability coinciding with slight off odour development^29^. Sensory scoring compared with storage time was adequately described by zero-order equations.

### Histological analysis

At the end of the trial period, three fish per tank, nine per diet group, were collected and killed with an overdose of MS 222 as previously described. Tissue samples, about 1 cm in length, from the anterior (just posterior to pyloric caeca) and posterior intestine (anterior to ileorectal valve) and liver were then removed and fixed in 10% phosphate buffered formalin. All samples were processed at increasing dehydration steps, cleared in xylene and embedded in paraffin wax, according to the standard procedures described by Bancroft and Gamble^24^. Longitudinal sections were cut at 5 μm thickness and stained with haematoxylin and eosin (H&E). Examination for any pathological alterations was performed using a light microscope at different magnifications. For the evaluation of the effects of the experimental diets on the microscopic anatomy of the liver and intestine, a simple ranking system was employed, based on the criteria presented in Supplementary Table S1.

### Statistical analysis

Tanks were considered as experimental units and fish represented the sample units. All data from the individual observations were tested for normality and homogeneity of variance prior to be subjected to one-way ANOVA using Kolmogorov–Smirnov and Levene’s tests, respectively. Tank means were used for comparisons. Significant differences between means were determined by Tukey’s test. The results from the organoleptic test panel were statistically evaluated by non-parametric Kruskal–Wallis test. The level of significance was set at *P* < 0.05.

## Results

### Growth and feeding efficiency

There was approximately a two-fold increase in body weight after the 12-week growth trial. Fish fed the C+ and T20 diets showed significantly higher values for weight gain (WG) and daily growth index (DGI) compared to the C− and T5 diets, while the opposite trend was detected for the thermal growth coefficient. Fish fed the T10 had WG and DGI values not significantly different than all other treatments (Table 2). The feed conversion ratio (FCR) and protein efficiency ratio (PER) were not significantly different among the treatments. Similarly, hepatosomatic and lipidosomatic indices were similar among the treatments (Table 2).

**Table 2:** Growth performance, feeding efficiencies and body indices of European seabass fed increasing levels of taurine after 12 weeks.

	Experimental diets				
	C+	C−	T5	T10	T20
Initial body weight (g)	78 ± 0.6	78 ± 0.4	77 ± 0.6	78 ± 0.5	78 ± 0.2
Survival	98.9 ± 2.8	87 ± 9.6	92 ± 3.5	88 ± 10.5	94 ± 5.3
Final Body weight (g)	169 ± 0.9^b^	161 ± 2.2^a^	160 ± 2.1^a^	166 ± 0.9^ab^	169 ± 2.7^b^
Weight gain (g)	91 ± 1.2^b^	84 ± 2.7^a^	83 ± 1.9^a^	88 ± 1.3^ab^	91 ± 2.7^b^
Daily growth index (%)	1.77 ± 0.02^b^	1.66 ± 0.05^a^	1.66 ± 0.03^a^	1.73 ± 0.02^ab^	1.76 ± 0.04^ab^
Total feed intake (g DM) per fish	124 ± 12.8	129 ± 15.1	115 ± 12.4	117 ± 15.5	113 ± 3.1
Relative Feed intake (% DM)	158.7 ± 16.3	166.2 ± 20.5	149.1 ± 15.9	151.0 ± 20.8	144.5 ± 3.8
Specific growth rate	1.09 ± 0.01	1.03 ± 0.03	1.03 ± 0.02	1.07 ± 0.01	1.08 ± 0.02
Feed conversion ratio (% DM)	1.36 ± 0.13	1.53 ± 0.22	1.38 ± 0.12	1.33 ± 0.16	1.25 ± 0.06
Protein efficiency ratio	1.70 ± 0.16	1.54 ± 0.42	1.64 ± 0.14	1.75 ± 0.20	1.84 ± 0.06
Thermal growth coefficient (× 1,000)	0.64 ± 0.01^b^	0.60 ± 0.02^a^	0.60 ± 0.01^a^	0.62 ± 0.01^ab^	0.64 ± 0.02^b^
Lipidosomatic index (%)	9.04 ± 0.75	9.51 ± 2.44	9.88 ± 0.24	9.63 ± 0.90	9.03 ± 1.12
Hepatosomatic index	2.09 ± 0.29	2.16 ± 0.23	2.23 ± 0.26	2.26 ± 0.21	2.48 ± 0.11

Row means not sharing the same superscript letters are significantly different (*P* < 0.05). Data are mean ± SD.

### Whole-body and fillet proximate composition and fillet amino acid profile

No significant differences were detected in the fish whole-body and muscle proximate composition among the treatments (Table 3). The fillet amino acid composition was similar among the treatments, except for aspartate, alanine, tyrosine and methionine concentrations, which were found to be significantly lower in T20 compared to T10 treatment. Regarding muscle concentration of taurine expressed also as a percentage of muscle protein in all the taurine supplemented treatments showed significantly higher values compared to the groups without supplementation (Table 3).

**Table 3:** Whole body composition and muscle composition of European seabass fed the experimental diets.

	Experimental diets				
	C+	C−	T5	T10	T20
**Whole body composition (g/kg fresh weight)**					
Moisture	627 ± 10	607 ± 21	637 ± 9	636 ± 9	633 ± 2
Protein	160 ± 5	171 ± 6	158 ± 3	165 ± 7	158 ± 5
Lipid	156 ± 9	160 ± 4	143 ± 10	154 ± 2	147 ± 10
Ash	36 ± 5	25 ± 9	40 ± 2	41 ± 10	38 ± 5
**Muscle composition (g/kg fresh weight)**					
Water	727 ± 1.27	741 ± 2.3	749 ± 1.8	752 ± 3.5	762 ± 13.0
Protein	224 ± 11.8	227 ± 17	214 ± 10.5	219 ± 14.1	215 ± 16.6
Lipid	34 ± 0.20	26 ± 0.10	28 ± 0.10	26 ± 3.20	25 ± 0.8
Ash	14 ± 0.70	14 ± 0.40	15 ± 0.20	15 ± 0.80	14 ± 0.30
**Amino acid composition of the fish fillets per treatment (g per kg muscle)**					
Histidine	4.5 ± 0.70	4.8 ± 1.1	5.0 ± 0.3	5.4 ± 0.4	4.9 ± 0.4
Taurine	2.8^a^ ± 0.34	2.3^a^ ± 0.30	4.6^b^ ± 0.20	5.2^b^ ± 0.26	5.3^b^ ± 0.66
Serine	8.6 ± 0.43	8.5 ± 0.60	9.1 ± 0.28	9.2 ± 0.49	8.6 ± 0.35
Arginine	13.6 ± 0.16	14.0 ± 0.51	13.9 ± 0.0.58	14.2 ± 00.82	13.4 ± 00.65
Glycine	11.1 ± 0.55	11.7 ± 0.20	11.3 ± 1.05	11.0 ± 0.55	10.1 ± 0.36
Asx	23.7^ab^ ± 0.78	24.3^ab^ ± 1.26	23.9^ab^ ± 0.50	24.8^b^ ± 0.57	22.6^a^ ± 0.57
Glx	33.8 ± 11.8	34.8 ± 01.59	34.2 ± 0.90	35.3 ± 1.21	32.4 ± 1.04
Threonine	10.0 ± 0.35	10.1 ± 0.43	10.1 ± 0.26	10.4 ± 0.28	9.7 ± 0.28
Alanine	13.5^ab^ ± 0.44	13.9^ab^ ± 0.52	14.0^ab^ ± 0.11	14.1^b^ ± 0.53	13.0^a^ ± 0.34
Proline	7.1 ± 0.14	7.5 ± 0.17	7.3 ± 0.30	7.1 ± 0.46	6.8 ± 0.32
Cystine	0.9 ± 0.32	0.8 ± 0.4	0.7 ± 0.0	0.9 ± 0.12	0.8 ± 0.10
Lysine	19.9 ± 0.74	20.2 ± 9.6	20.3 ± 0.5	21.0 ± 0.50	19.4 ± 0.87
Tyrosine	7.7^ab^ ± 0.30	7.8^ab^ ± 0.41	7.7^ab^ ± 0.17	8.1^b^ ± 0.19	7.4^a^ ± 0.17
Methionine	6.9^ab^ ± 0.31	7.0^ab^ ± 0.36	6.9^ab^ ± 0.05	7.1^b^ ± 0.17	6.6^a^ ± 0.15
Valine	10.3 ± 0.38	10.5 ± 0.51	10.4 ± 0.40	11.0 ± 0.20	10.1 ± 0.23
Isoleucine	9.6 ± 0.40	9.7 ± 6.4	9.9 ± 0.28	10.3 ± 0.22	9.4 ± 0.25
Leucine	17.5 ± 0.63	17.8 ± 0.92	17.7 ± 0.46	18.4 ± 0.41	16.9 ± 0.00.47
Phenylalanine	9.2 ± 0.32	9.4 ± 0.50	9.4 ± 0.10	9.6 ± 0.17	8.8 ± 0.20
Tau (%)*	0.125^a^ ± 0.015	0.102^a^ ± 0.013	0.213^b^ ± 0.010	0.233^b^ ± 0.0134	0.248^b^ ± 0.031

Data are mean ± SD. Column means not sharing the same superscript letters are significantly different (*P* < 0.05).

*Expressed as percentage of muscle protein.

### Texture and sensory analysis

**Figure 1:**
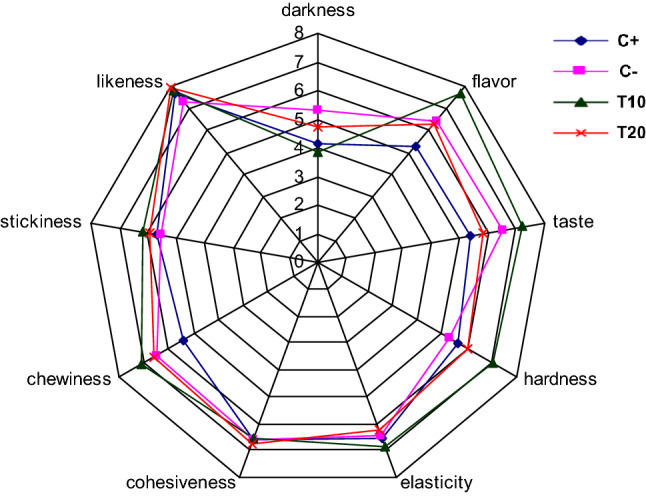
Descriptive taste panel results of fillets from seabass fed the experimental diets.

The hardness and chewiness of the fillets from seabass were significantly higher in fish fed the T10 or T20 diets compared to the other treatments, while adhesiveness was significantly lower in fish fed the T10 or T20 diets (Table 4). The results from test panel showed that seabass fillets had no significant differences in darkness, taste, elasticity, cohesiveness, chewiness or likeness among the dietary treatments. However, the flavor value was significantly higher in the T10 treatment compared to C+. Similarly, the hardness value was significantly higher in the T10 treatment compared to the C+ and C− treatments (Table 4; Fig. 1).

**Table 4:** Texture parameters and descriptive taste panel results of the fillet from seabass fed different experimental diets.

	Texture parameters of the fillet			Descriptive taste panel results							
	Hardness	Chewiness	Adhesiveness	Darkness	Flavor	Taste	Hardness	Elasticity	Cohesiveness	Chewiness	Likeness
C+	1.72 ± 0.10 ^a^	0.73 ± 0.04 ^a^	-0.031 ± 0.005 ^ab^	4.16 ± 3.18	5.32 ± 2.65^a^	5.42 ± 2.60	5.63 ± 2.14^a^	6.52 ± 2.52	6.58 ± 2.55	5.42 ± 2.47	5.68 ± 1.80
C−	2.05 ± 0.05 ^a^	0.56 ± 0.07 ^a^	-0.018 ± 0.002 ^a^	5.33 ± 3.39	6.42 ± 2.29^ab^	6.53 ± 2.57	5.26 ± 2.36^a^	6.42 ± 2.58	6.58 ± 2.50	6.47 ± 2.57	5.58 ± 2.38
T10	2.65 ± 0.10 ^b^	0.79 ± 0.08 ^ab^	-0.050 ± 0.010 ^bc^	3.83 ± 3.04	7.68 ± 2.68^b^	7.21 ± 2.90	7.00 ± 2.27^b^	6.84 ± 2.70	6.53 ± 2.48	7.05 ± 2.88	6.21 ± 2.41
T20	3.73 ± 0.19 ^c^	1.07 ± 0.17 ^b^	-0.063 ± 0.009 ^c^	4.75 ± 3.13	6.32 ± 2.65^ab^	5.84 ± 2.33	6.00 ± 2.12^ab^	6.26 ± 2.62	6.74 ± 2.24	6.58 ± 2.83	5.95 ± 1.78

Columns means not sharing the same superscript letters are significantly different (*P* < 0.05). Data are means ± SD.

### Cultivable bacteria and total volatile basic nitrogen

**Figure 2:**
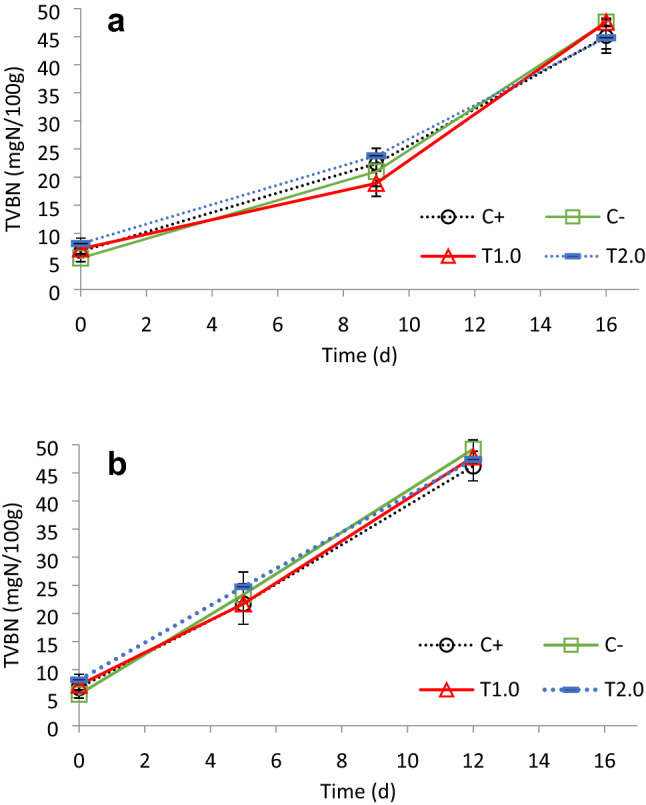
Total volatile basic nitrogen (TVBN) changes (mg N/100 g) (mean ± SD) of seabass fillets packed aerobically and stored at 0 °C (**a**) or 5 °C (**b**) after being fed the experimental diets.

Growth curves of total viable count, *Pseudomonas* spp. and *Enterobacteriaceae* spp. in fish fillets stored at 0 and 5 °C were fitted to the Baranyi equation and the growth kinetic parameters at each condition were determined. All the exponential growth rates of the measured microorganisms, in the various conditions are presented in Table 5, showing no significant (*P* > 0.05) differences between the different diets. No significant dietary effect was detected on the amount of total volatile basic nitrogen (TVBN) at either 0 °C or 5 °C (Fig. 2a, b). The TVBN values were significantly higher at 5 °C compared to at 0 °C.

**Table 5:** Exponential growth rates (d^−1^) of total viable count, *Pseudomonas* spp. and *Enterobacteriaceae* spp. and shelf life of seabass fillets stored aerobically at 0 and 5 °C (mean values ± standard deviation based on the statistical variation of the kinetic parameters of the Baranyi growth model—regression analysis).

	Total viable count		*Pseudomonas* spp.		*Enterobacteriaceae* spp.		*Shelf life*	
	0 °C	5 °C	0 °C	5 °C	0 °C	5 °C	0 °C	5 °C
C+	0.47 ± 0.15	0.53 ± 0.23	0.51 ± 0.13	0.60 ± 0.19	0.56 ± 0.25	0.89 ± 0.08	12d	5d
C−	0.43 ± 0.12	0.46 ± 0.12	0.48 ± 0.15	0.59 ± 0.10	0.47 ± 0.11	0.78 ± 0.01	11d	6d
T10	0.43 ± 0.09	0.48 ± 0.22	0.49 ± 0.08	0.68 ± 0.12	0.59 ± 0.13	0.85 ± 0.01	12d	6d
T20	0.45 ± 0.08	0.46 ± 0.17	0.51 ± 0.06	0.59 ± 0.24	0.51 ± 0.16	0.74 ± 0.10	11d	5d

Columns means not sharing the same superscript letters are significantly different (*P* < 0.05). Data are mean ± SD.

### Drip loss and self-life

**Figure 3:**
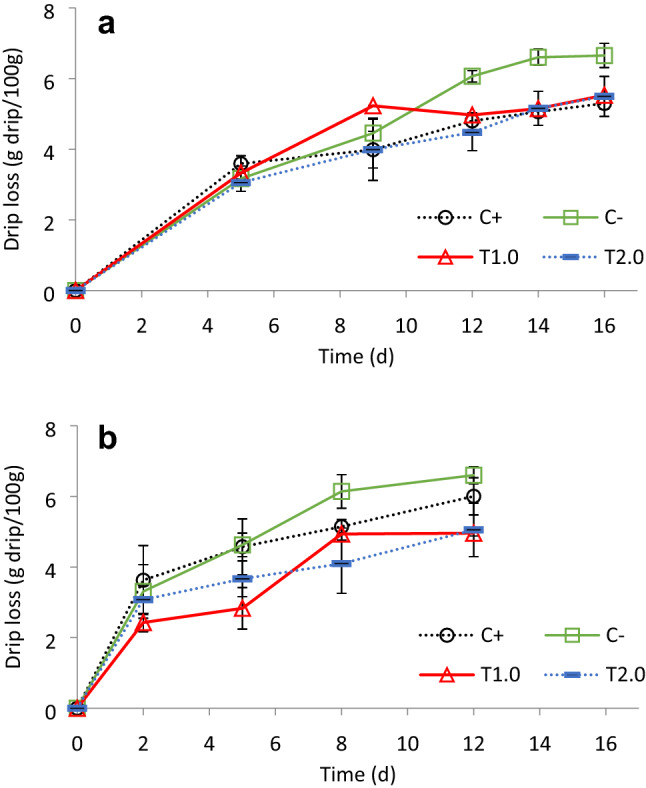
Drip loss (g drip/100 g) (± SD) of seabass fillets packed aerobically and stored at 0 °C (**a**) or 5 °C (**b**) for fish fed the experimental diets. Asterisks indicates significant differences (*P* < 0.05) between C− and the T1.0 and T2.0% treatments.

Fillets of fish fed the C+ diet had a significantly higher drip loss value at 0 °C (Fig. 3a), but no significant differences were detected at 5 °C among the different dietary treatments (Fig. 3b). No significant difference was detected among the other treatments at either temperature (Fig. 3a, b). Regarding the self-life of fillets initially were slightly translucent and had a sweet seaweed-like odour, but gradually developed into a strong fishy odour. A lower temperature of 0 °C doubled the shelf-life of all fillets compared to at 5 °C, however, no significant differences were detected among the treatments (Table 5).

### Histological analysis

**Figure 4:**
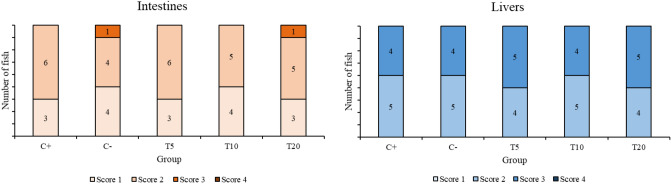
Scoring of seabass intestine and liver tissue from the different dietary treatments.

The microscopic structure of the intestine samples appeared similar among the treatments and overall normal, though in many samples focal hyperplastic enteritis was observed in some intestinal folds (Supplementary Fig. S1). In each fish, similar findings were observed between the two intestine segments, thus the scoring presented in Fig. 4 reflects the evaluation of both segments per fish. Furthermore, no clear pattern was observed among the treatments. All liver samples exhibited mild to moderate vacuolation of the hepatocytes, possibly due to fat accumulation within the cytoplasm (Fig. 4 and Supplementary Fig. S2).

## Discussion

Our study sufficiently demonstrated the fundamental role of taurine in mediating the growth of European seabass juveniles. In fact, taurine supplementation at 20 g/kg in a high plant—low FM diet (15%) significantly improved the growth of European seabass juveniles compared to those fed the non-supplemented diet. Particularly, noteworthy was the finding that taurine addition at higher inclusion levels (10 and 20 g/kg) in the low FM diet led to a similar growth performance to the control diet, which contained a high FM inclusion (30%).

The beneficial effect of taurine supplementation in low FM diets on growth performance and feed utilization of European seabass fry and juveniles has been also reported in previous studies^15,31,35^. Although taurine can act as a feeding stimulant^32^, this was unlikely the cause in this study based on the observed similar feed intake among all treatments. This has similarly been suggested previously in European seabass fed taurine supplemented diets^16,33^. Moreover, the taurine content of T20 diet was far exceeded that of the C+ diet, indicating that the growth improvement is unrelated to prevent a taurine deficiency. On the other hand, the growth promoting effects of taurine have been linked to regulation of metabolism and improved nutrient utilization/feeding efficiency^6,16^.

One factor that may improve nutrient utilization in fish fed taurine supplemented diets includes beneficial histological characteristics of the intestine. A study contacted by Tian et al.^34^ found that the dietary taurine from 1 − 4 g/kg led to longer intestinal folds, and thus increased surface area for nutrient absorption, in black carp, *Mylopharyngodon piceus*, juveniles. Few previous studies have examined the effects of taurine supplementation on seabass intestine histology. Martins et al.^33^ observed no significant changes in the anterior intestine of seabass, when fed diet supplemented with 1% taurine. In the posterior intestine however, taurine supplementation reduced the signs of inflammation caused by the increased level of plant protein mix, and specifically resulted in an decrease in the intraepithelial lymphocytes number, improvement of nucleus position within the enterocytes and decreased enterocyte vacuolation. In another study, Rimoldi et al.^35^, did not observe any significant differences in the overall morphology of the intestine of seabass when fed feed with 0.2% taurine, though they focused mostly on the development of the muscle layer. However, they observed some differences in the microvilli in the group fed the taurine supplemented diet (more developed), using electron microscopy. In the present study, based on the histological scoring system used, while the fish did show some instances of localized intestinal enteritis in all treatments, taurine supplementation did not appear to improve intestinal health. This discrepancy is not surprising considering the contrasting findings regarding the feasibility of replacing FM with plant proteins in the diets of European seabass. For example, Kaushik et al.^36^ reported that 95% of FM can be replaced with various plant proteins (soybean meal, corn gluten meal and rapeseed meal) without negatively affecting their performance. In contrast, others showed depressed growth when using similar blends of various plant proteins^33,37,38^. The possible explanation for such inconsistencies might be the differences in rearing systems, raw materials, feeding methods, possible genetic differences in the fish used etc.

In the current study, taurine supplementation had no observable effects, either beneficial or negative, to the livers of European seabass, which is in agreement with a previous study conducted by Kotzamanis et al.^17^ using moderate replacement of FM and taurine supplementation*.* In contrast, López et al.^39^ found that totoaba, *Totoaba macdonaldi*, juveniles had less abnormal liver histopathology when taurine (10 g/kg) was added in soy protein concentrate—based diets compared to without added taurine. Further investigation is required to better understand these contrasting findings.

Muscle taurine levels largely mirrored those in the diet, which has been also reported in other species^9,17,40,41^. However, it should be noted that a plateau was found in muscle taurine concentration between the T10 and T20 treatments probably due to a saturation in its deposition. Nevertheless, this led to no significant changes to the whole-body proximate composition, as observed in cobia, *Rachycentron canadum*^42^, totoaba, *Totoaba macdonaldi*^38^ and European seabass^14,17,33^.

Notably, other post-harvest characteristics that are not often investigated were found to be significantly affected in the present study, which can be a substantial factor to consumer acceptability and thus market demand^17^. The blind taste panel found significant differences in flavour and hardness in the fillets of European seabass fed the T10 diet, a finding which was not detected in a previous study of Kotzamanis et al.^17^ using a moderate FM inclusion (25%). This seems more likely as a result of lower drip loss, which is defined as the loss of liquids after thawing meat. Such liquid originates from cell rupture and contains nutrients and other flavourful compounds, and thus reducing drip loss is desired. Lu et al.^43^ reported that taurine supplementation significantly decreased the drip loss of breast meat in chronic heat-stressed broilers, but our finding appears to be the first reported for a fish species. Taurine is well known to improve the structural integrity of cell membranes by interacting with the phospholipids^44^ or protecting cell membrane lipids from oxidative destruction by scavenging reactive oxygen species (ROS) in fish liver and muscle^31,45^ and this may be a major contributor to taurine reducing drip loss. Moreover, taurine deficiency in fish is responsible for causing “green liver syndrome” due to osmotically fragile hepatocytes that would lyse and release excessive green bile pigments (e.g. biliverdin and bilirubin)^46^. This is supported by other studies in which taurine supplementation prevented “green liver syndrome” in yellowtail (*Seriola quinqueradiata*)^47^ and totoaba (*Totoaba macdonaldi*)^48^. Moreover, our findings showed that the texture parameters of European seabass fillets were also improved by taurine supplementation by means of increasing fillet hardness and chewiness in a linear and polynomial pattern, respectively. A firmer texture is preferable, and it is considering an indicator of freshness^49^. Some amino acids, such as glutamate and/or arginine, can increase muscle cell density as well as the connective tissue leading to a harder texture^50,51^. While this may have similarly occurred in European seabass, it could not be confirmed based on the histological examination of the overall muscle structure and cells. However, it is important to note that histology was performed on newly deceased fish, and not on those after storage when connective tissue degradation mostly occurs.

In addition to texture and flavour, the smell of fish fillets is a characteristic for consumers to judge product freshness. Some of the factors that influence smell include the growth of spoilage bacteria that produce various off—flavors as well as the breakdown of protein that produces total volatile basic nitrogen (TVBN)^52^. In the present study, taurine had no effect on either spoilage bacterial growth or TVBN levels, indicating that fillet shelf-life was unaffected by feeding European seabass with dietary taurine supplementation. Indeed, taurine is not known to have antimicrobial properties, which likely explains this finding. Further research is required to understand the role of taurine on improving fish growth performance and sensory characteristics of fillet.

## Conclusions

This study showed that taurine supplementation of 20 g/kg in plant-based diets substantially improved the growth of European seabas*s* juveniles, which can potentially have an economical advantage over the FM-based diets. Moreover, the taurine supplemented diets enhanced the sensory characteristics of fish fillet. It appears this was likely due to taurine increasing fillet hardness and minimizing drip loss, thus maintaining the fillet flavor. This finding suggests that taurine may reduce incidences of cell lysis during the thawing process, rather than taurine itself being a flavour enhancer or minimizing some factors that contribute to off-flavors. In the case of the latter, these are often caused from the growth of spoilage bacteria and production of TVBN, but taurine had no effect on these parameters. Nevertheless, high taurine supplementation can be strongly recommended as an effective way to improve growth in European seabass fed high plant protein diets as well as to improve the organoleptic properties of the fillet.

## Supplementary information

Supplementary Information.
