# Supplementary material for: High taurine supplementation in plant protein-based diets improves growth and organoleptic characteristics of European seabass (Dicentrarchus labrax)
Source: Sci Rep. 2020 Jul 23;10:12294. doi: 10.1038/s41598-020-69014-x (PMC7378086; doi:10.1038/s41598-020-69014-x)
Supplement: Supplementary file 1 — Supplementary Information. [file 41598_2020_69014_MOESM1_ESM.docx]

**SUPPLEMENTARY FILE**

# High taurine supplementation in plant protein-based diets improves growth and organoleptic characteristics of European seabass (*Dicentrarchus labrax*)

Yannis Kotzamanis ^1^*, Theofania Tsironi ^2^, Andreas Brezas ^3^, Kriton Grigorakis ^1^, Vassiliki Ilia^1^, Ioannis Vatsos^4^, Nicholas Romano^5^, Jan van Eys^6^, Vikas Kumar^3^*

^1^Hellenic Centre for Marine Research (HCMR) Institute of Marine Biology, Biotechnology and Aquaculture, Fish Nutrition Lab, Agios Kosmas, Hellinikon, 16777 Athens, Greece

^2^Agricultural University of Athens, Department of Food Science and Human Nutrition, Food Process Engineering Laboratory, Athens, 11855, Greece

^3^Aquaculture Research Institute, Department of Animal and Veterinary Science, University of Idaho, Moscow, ID 83844, USA

^4^Faculty of Biosciences and Aquaculture, Nord University, Post Box 1490, 8049 Bodø, Norway

^5^ Department of Aquaculture and Fisheries, University of Arkansas at Pine Bluff, USA

^6^ GANS Inc. 24 Av. de la Guillemotte, 78112, Fourqueux, France

***Corresponding authors**: jokotz@hcmr.gr and vikaskumar@uidaho.edu

**Supplementary Table S1.** Criteria used for the histological evaluation of the intestine and liver of seabass at the end of feeding trial.

| **Intestine** | |
| --- | --- |
| **Score** | **Description** |
| **1** (normal) | Intestinal folds are long, with obvious indentations, thin lamina propria and submucosa. Occasional areas of thickening of the lamina propria and submucosa. |
| **2** (mild enteritis) | Few areas with loss of indentation of the folds and increased width of the lamina propria and submucosa. |
| **3** (moderate enteritis) | Many areas with loss of indentation of the folds and increased width of the lamina propria and submucosa. |
| **4** (severe enteritis) | Marked reduction in the height of the intestinal folds, with severe thickening of the lamina propria and submucosa. Extensive loss of normal appearance of the intestinal epithelium |
| **Liver** | |
| **Score** | **Description** |
| **1** (normal) | The hepatocytes are small, with few small vacuoles in their cytoplasm, central positioning of their nucleus. |
| **2** (mild vacuolation) | The hepatocytes are slightly enlarged, with many vacuoles in the cytoplasm, nucleus is positioned centrally in most hepatocytes. |
| **3** (moderate vacuolation) | All hepatocytes are enlarged, with many vacuoles in their cytoplasm, or one big vacuole and their nucleus is pushed towards the periphery. |
| **4** (severe vacuolation and inflammation) | Almost all hepatocytes are significantly enlarged, the cytoplasm appears ‘empty’ due to the presence of big vacuoles. The nucleus of many hepatocytes appears pyknotic. In a few cases, the hepatocytes appear broken and areas of cell necrosis can be seen. |


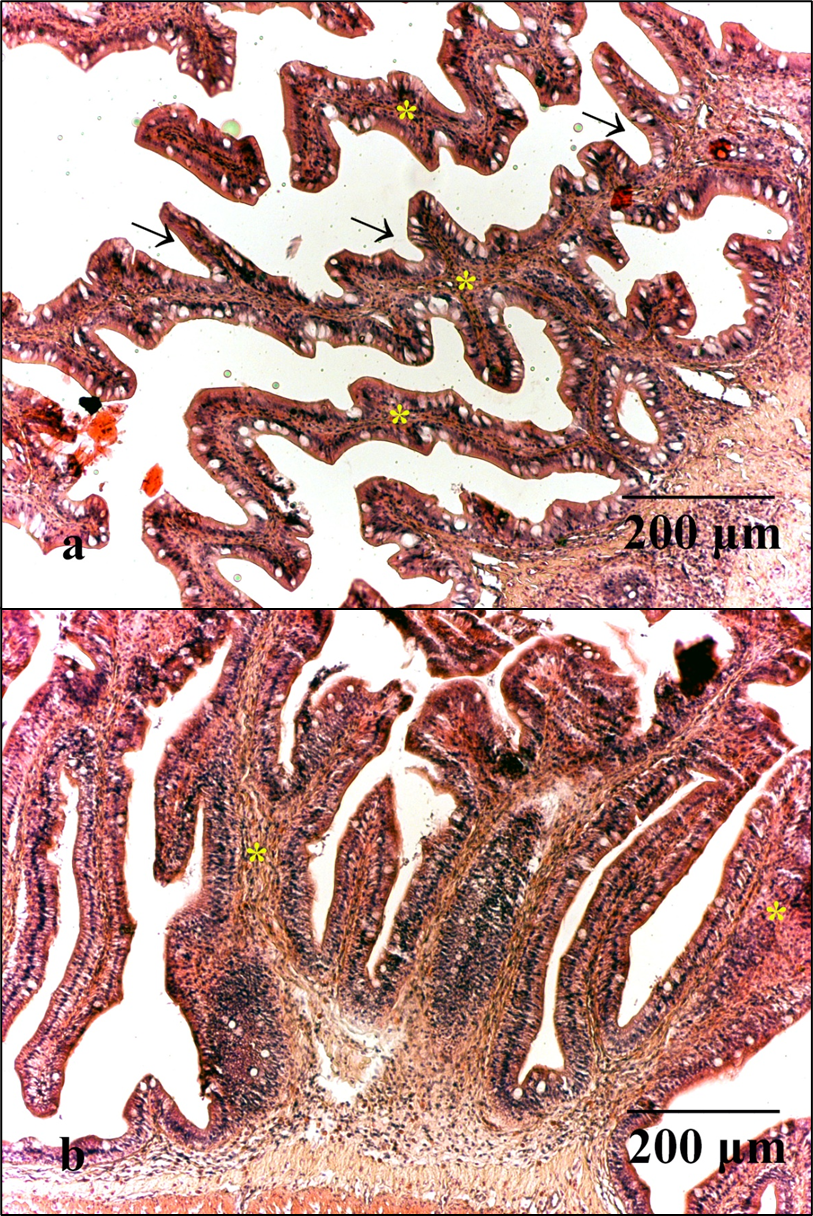


**Supplementary Fig. S1.** Seabass intestine tissue sections a) anterior intestine - sample collected from the T10 group exhibiting score 1: the intestinal folds appear long with good mucosal indentations (arrows) and thin lamina propria (*) b) anterior intestine - sample collected from the T5 group, exhibiting score 2: an area where intestinal folds are long but there is loss of mucosal indentations and the lamina propria appears thickened in many areas (*). Stain H&E.


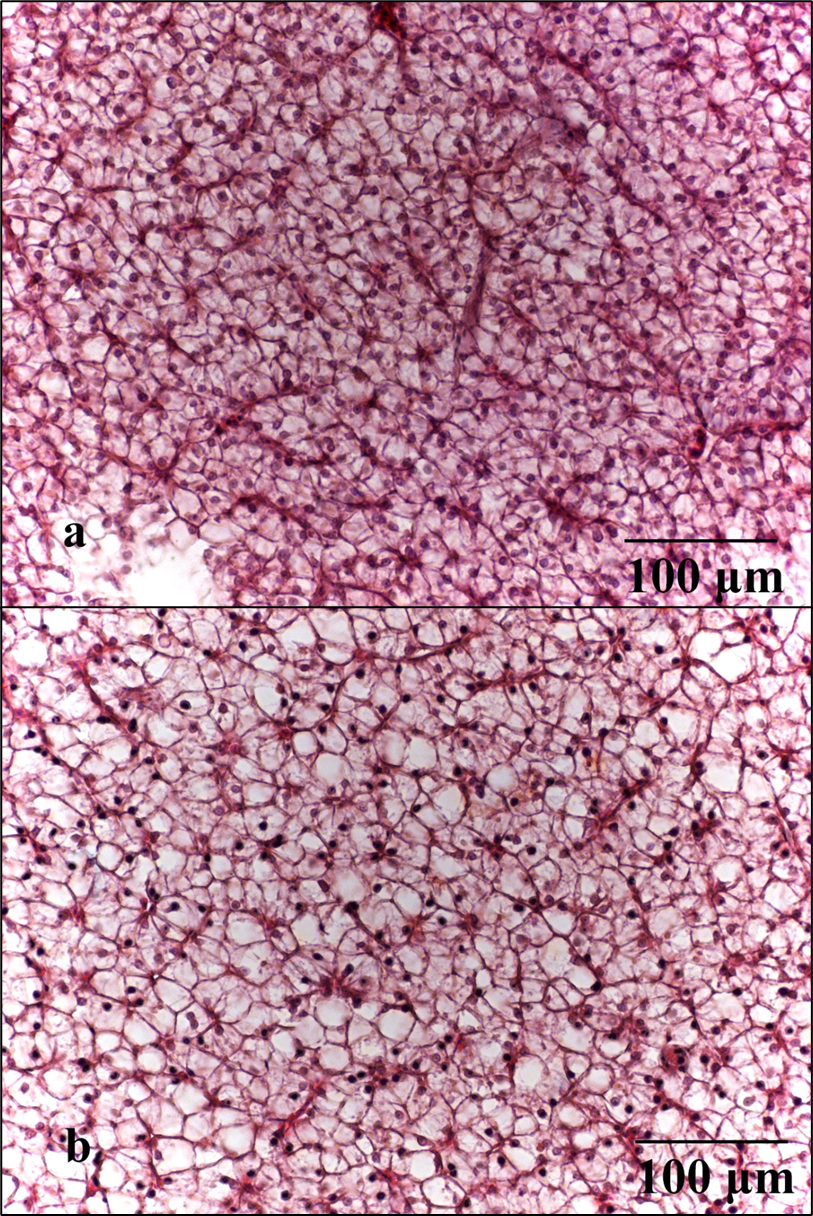


**Supplementary Fig. S2.** Seabass liver tissue sections a) sample collected from the C+ group exhibiting score 2: all hepatocytes appear to contain vacuoles, but their nuclei are located mostly at the center. b) sample from T20 group, exhibiting score 3: there is notable increase in the size of the hepatocytes due to the vacuolation and their nuclei are mostly displaced towards the periphery. Stain H&E.
